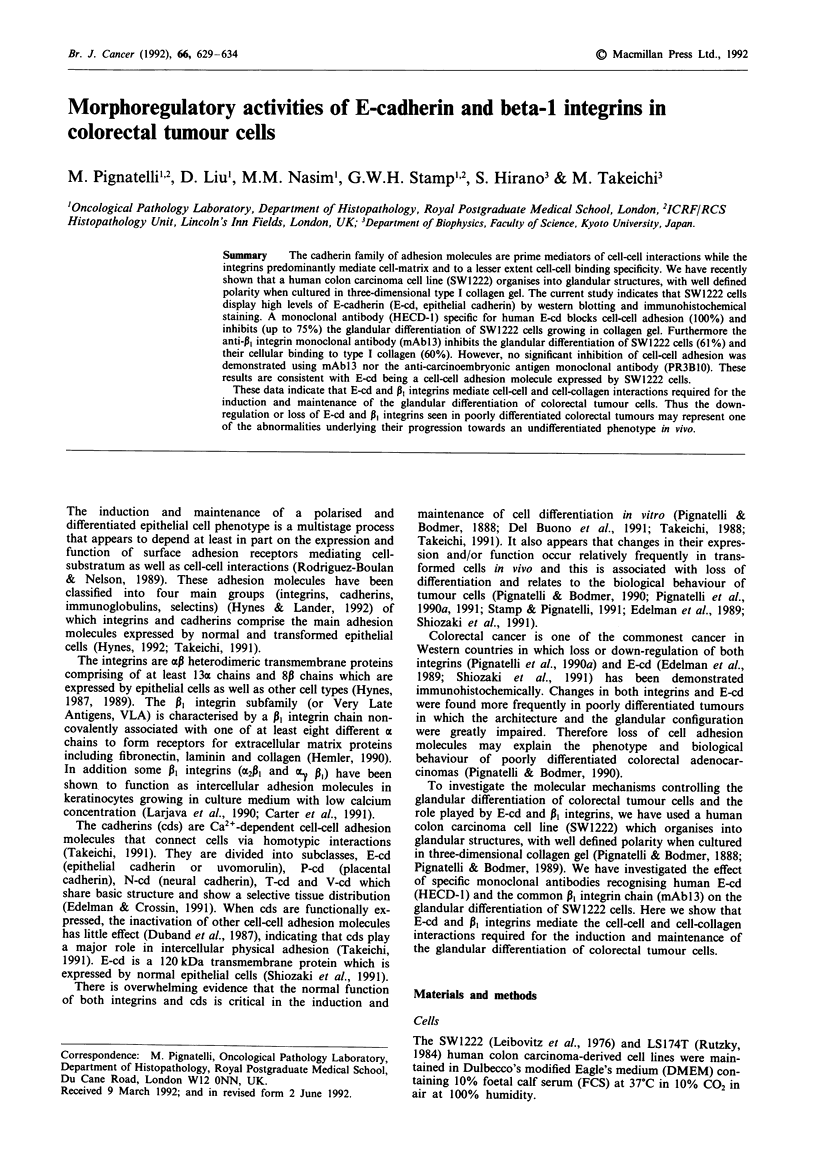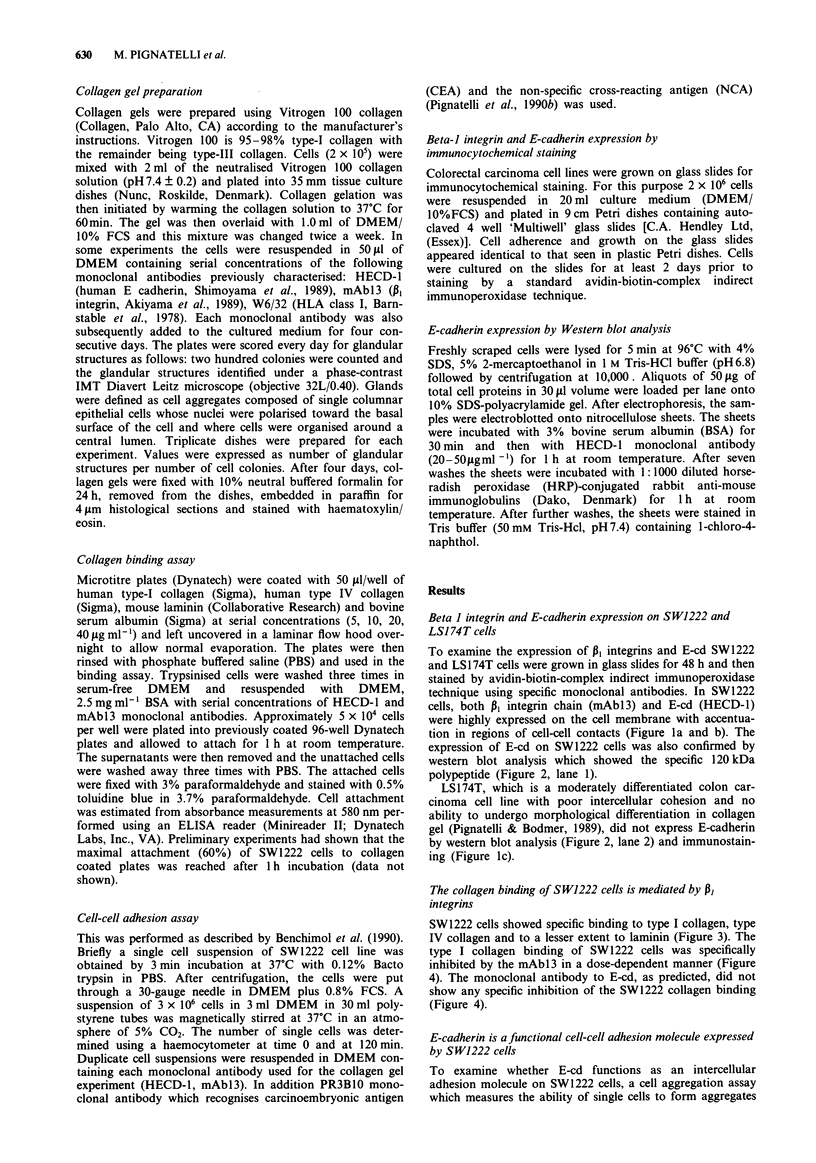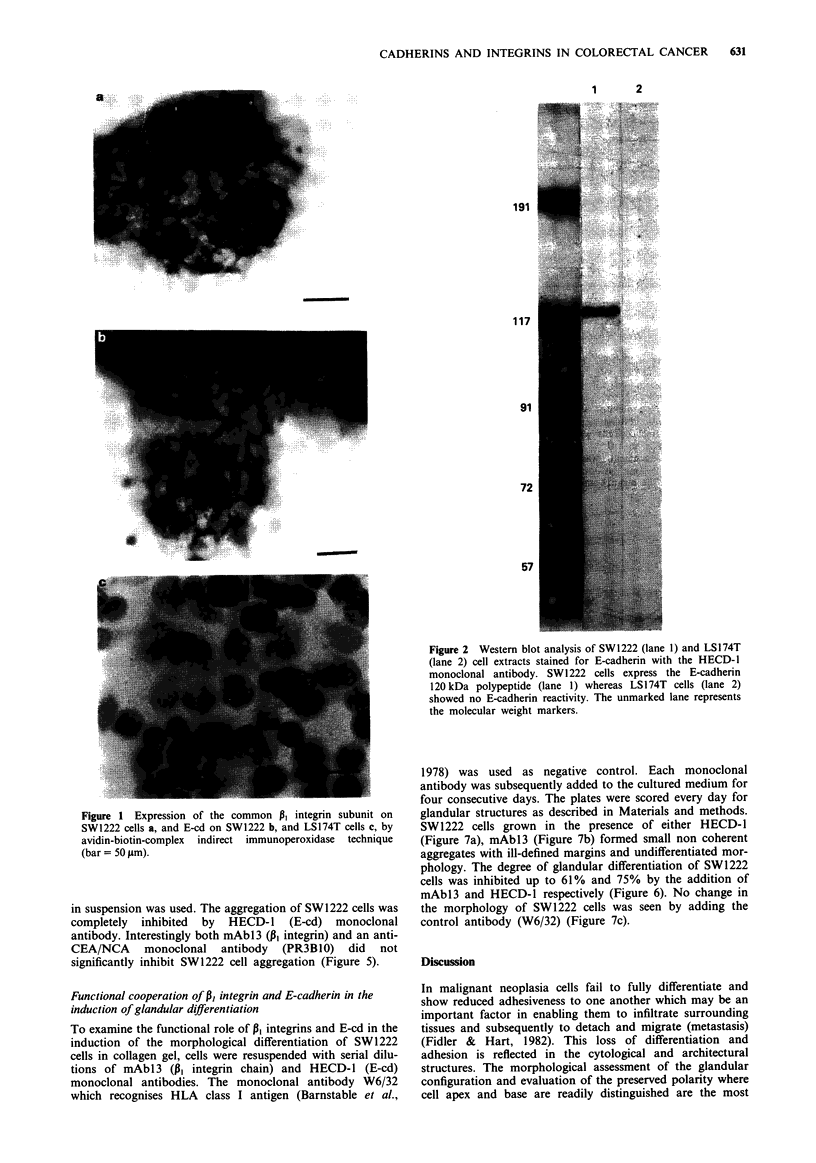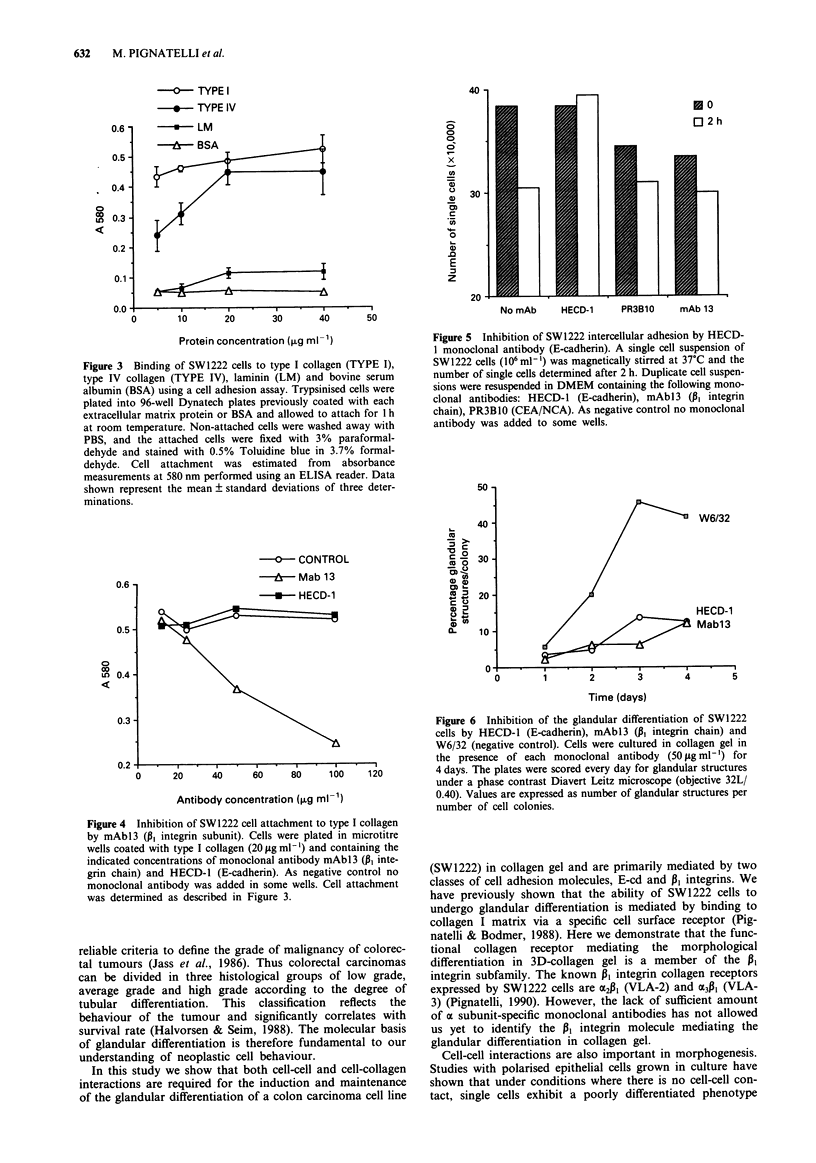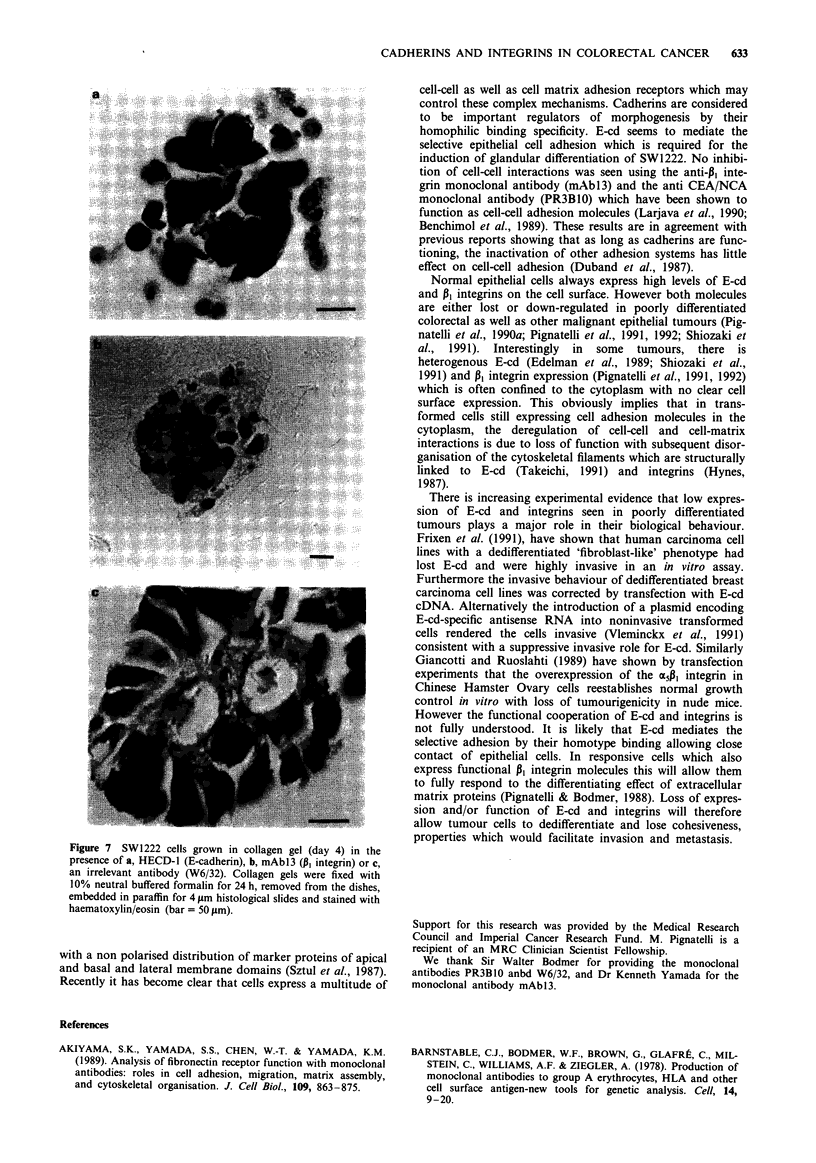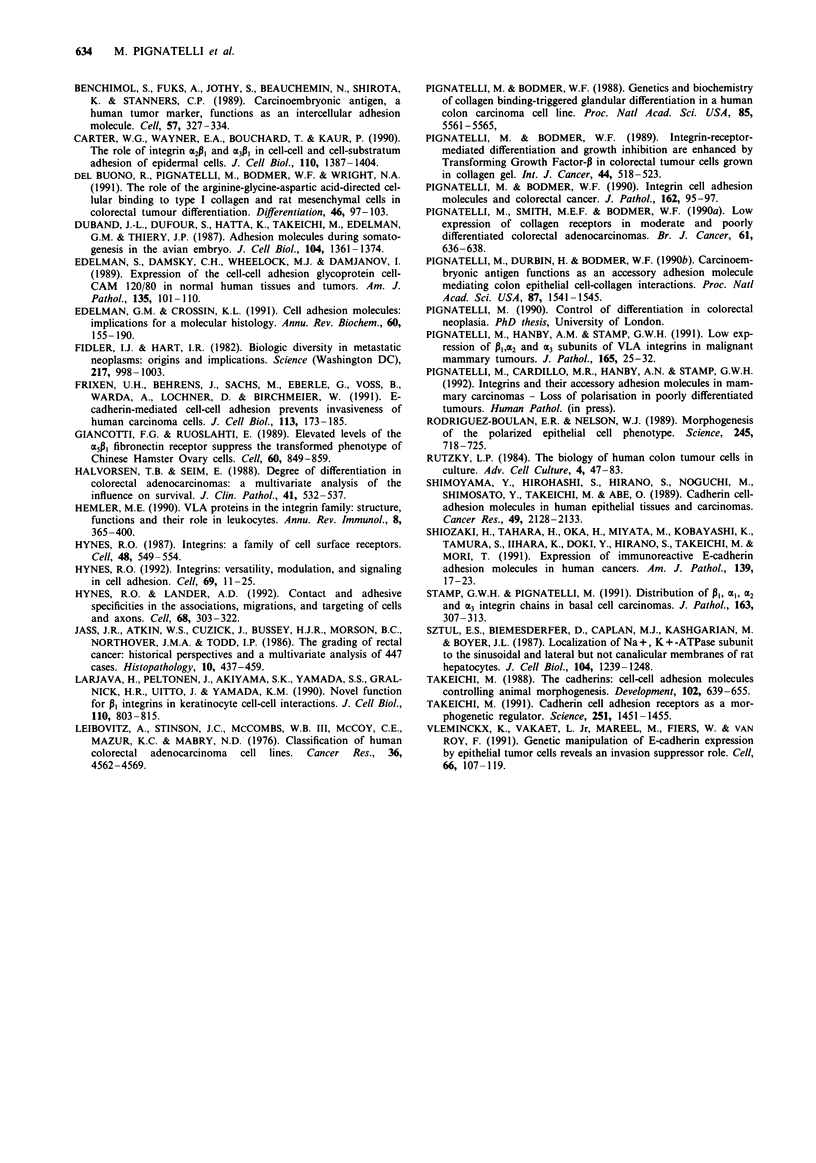# Morphoregulatory activities of E-cadherin and beta-1 integrins in colorectal tumour cells.

**DOI:** 10.1038/bjc.1992.328

**Published:** 1992-10

**Authors:** M. Pignatelli, D. Liu, M. M. Nasim, G. W. Stamp, S. Hirano, M. Takeichi

**Affiliations:** Department of Histopathology, Royal Postgraduate Medical School, London, UK.

## Abstract

**Images:**


Br. J. Cancer (1992), 66, 629–634 © Macmillan Press Ltd., 1992

### Morphoregulatory activities of E-cadherin and beta-1 integrins in colorectal tumour cells

M. Pignatelli[1,2], D. Liu[1], M.M. Nasim[1], G.W.H. Stamp[1,2], S. Hirano[3] & M. Takeichi[3]

[1]Oncological Pathology Laboratory, Department of Histopathology, Royal Postgraduate Medical School, London, [2]ICRF/RCS Histopathology Unit, Lincoln's Inn Fields, London, UK; [3]Department of Biophysics, Faculty of Science, Kyoto University, Japan.

**Summary** The cadherin family of adhesion molecules are prime mediators of cell-cell interactions while the integrins predominantly mediate cell-matrix and to a lesser extent cell-cell binding specificity. We have recently shown that a human colon carcinoma cell line (SW1222) organises into glandular structures, with well defined polarity when cultured in three-dimensional type I collagen gel. The current study indicates that SW1222 cells display high levels of E-cadherin (E-cd, epithelial cadherin) by western blotting and immunohistochemical staining. A monoclonal antibody (HECD-1) specific for human E-cd blocks cell-cell adhesion (100%) and inhibits (up to 75%) the glandular differentiation of SW1222 cells growing in collagen gel. Furthermore the anti-$\beta_1$ integrin monoclonal antibody (mAb13) inhibits the glandular differentiation of SW1222 cells (61%) and their cellular binding to type I collagen (60%). However, no significant inhibition of cell-cell adhesion was demonstrated using mAb13 nor the anti-carcinoembryonic antigen monoclonal antibody (PR3B10). These results are consistent with E-cd being a cell-cell adhesion molecule expressed by SW1222 cells.

These data indicate that E-cd and $\beta_1$ integrins mediate cell-cell and cell-collagen interactions required for the induction and maintenance of the glandular differentiation of colorectal tumour cells. Thus the down-regulation or loss of E-cd and $\beta_1$ integrins seen in poorly differentiated colorectal tumours may represent one of the abnormalities underlying their progression towards an undifferentiated phenotype *in vivo*.

The induction and maintenance of a polarised and differentiated epithelial cell phenotype is a multistage process that appears to depend at least in part on the expression and function of surface adhesion receptors mediating cell-substratum as well as cell-cell interactions (Rodriguez-Boulan & Nelson, 1989). These adhesion molecules have been classified into four main groups (integrins, cadherins, immunoglobulins, selectins) (Hynes & Lander, 1992) of which integrins and cadherins comprise the main adhesion molecules expressed by normal and transformed epithelial cells (Hynes, 1992; Takeichi, 1991).

The integrins are $\alpha\beta$ heterodimeric transmembrane proteins comprising of at least 13$\alpha$ chains and 8$\beta$ chains which are expressed by epithelial cells as well as other cell types (Hynes, 1987, 1989). The $\beta_1$ integrin subfamily (or Very Late Antigens, VLA) is characterised by a $\beta_1$ integrin chain non-covalently associated with one of at least eight different $\alpha$ chains to form receptors for extracellular matrix proteins including fibronectin, laminin and collagen (Hemler, 1990). In addition some $\beta_1$ integrins ($\alpha_2\beta_1$ and $\alpha_\gamma$ $\beta_1$) have been shown to function as intercellular adhesion molecules in keratinocytes growing in culture medium with low calcium concentration (Larjava *et al.*, 1990; Carter *et al.*, 1991).

The cadherins (cds) are $Ca^{2+}$-dependent cell-cell adhesion molecules that connect cells via homotypic interactions (Takeichi, 1991). They are divided into subclasses, E-cd (epithelial cadherin or uvomorulin), P-cd (placental cadherin), N-cd (neural cadherin), T-cd and V-cd which share basic structure and show a selective tissue distribution (Edelman & Crossin, 1991). When cds are functionally expressed, the inactivation of other cell-cell adhesion molecules has little effect (Duband *et al.*, 1987), indicating that cds play a major role in intercellular physical adhesion (Takeichi, 1991). E-cd is a 120 kDa transmembrane protein which is expressed by normal epithelial cells (Shiozaki *et al.*, 1991).

There is overwhelming evidence that the normal function of both integrins and cds is critical in the induction and maintenance of cell differentiation *in vitro* (Pignatelli & Bodmer, 1888; Del Buono *et al.*, 1991; Takeichi, 1988; Takeichi, 1991). It also appears that changes in their expression and/or function occur relatively frequently in transformed cells *in vivo* and this is associated with loss of differentiation and relates to the biological behaviour of tumour cells (Pignatelli & Bodmer, 1990; Pignatelli *et al.*, 1990*a*, 1991; Stamp & Pignatelli, 1991; Edelman *et al.*, 1989; Shiozaki *et al.*, 1991).

Colorectal cancer is one of the commonest cancer in Western countries in which loss or down-regulation of both integrins (Pignatelli *et al.*, 1990*a*) and E-cd (Edelman *et al.*, 1989; Shiozaki *et al.*, 1991) has been demonstrated immunohistochemically. Changes in both integrins and E-cd were found more frequently in poorly differentiated tumours in which the architecture and the glandular configuration were greatly impaired. Therefore loss of cell adhesion molecules may explain the phenotype and biological behaviour of poorly differentiated colorectal adenocarcinomas (Pignatelli & Bodmer, 1990).

To investigate the molecular mechanisms controlling the glandular differentiation of colorectal tumour cells and the role played by E-cd and $\beta_1$ integrins, we have used a human colon carcinoma cell line (SW1222) which organises into glandular structures, with well defined polarity when cultured in three-dimensional collagen gel (Pignatelli & Bodmer, 1888; Pignatelli & Bodmer, 1989). We have investigated the effect of specific monoclonal antibodies recognising human E-cd (HECD-1) and the common $\beta_1$ integrin chain (mAb13) on the glandular differentiation of SW1222 cells. Here we show that E-cd and $\beta_1$ integrins mediate the cell-cell and cell-collagen interactions required for the induction and maintenance of the glandular differentiation of colorectal tumour cells.

### Materials and methods

### Cells

The SW1222 (Leibovitz *et al.*, 1976) and LS174T (Rutzky, 1984) human colon carcinoma-derived cell lines were maintained in Dulbecco's modified Eagle's medium (DMEM) containing 10% foetal calf serum (FCS) at 37°C in 10% $CO_2$ in air at 100% humidity.

Correspondence: M. Pignatelli, Oncological Pathology Laboratory, Department of Histopathology, Royal Postgraduate Medical School, Du Cane Road, London W12 0NN, UK.
Received 9 March 1992; and in revised form 2 June 1992.

*Collagen gel preparation*

Collagen gels were prepared using Vitrogen 100 collagen (Collagen, Palo Alto, CA) according to the manufacturer's instructions. Vitrogen 100 is 95–98% type-I collagen with the remainder being type-III collagen. Cells ($2 \times 10^5$) were mixed with 2 ml of the neutralised Vitrogen 100 collagen solution (pH 7.4 ± 0.2) and plated into 35 mm tissue culture dishes (Nunc, Roskilde, Denmark). Collagen gelation was then initiated by warming the collagen solution to 37°C for 60 min. The gel was then overlaid with 1.0 ml of DMEM/10% FCS and this mixture was changed twice a week. In some experiments the cells were resuspended in 50 µl of DMEM containing serial concentrations of the following monoclonal antibodies previously characterised: HECD-1 (human E cadherin, Shimoyama *et al.*, 1989), mAb13 ($\beta_1$ integrin, Akiyama *et al.*, 1989), W6/32 (HLA class I, Barnstable *et al.*, 1978). Each monoclonal antibody was also subsequently added to the cultured medium for four consecutive days. The plates were scored every day for glandular structures as follows: two hundred colonies were counted and the glandular structures identified under a phase-contrast IMT Diavert Leitz microscope (objective 32L/0.40). Glands were defined as cell aggregates composed of single columnar epithelial cells whose nuclei were polarised toward the basal surface of the cell and where cells were organised around a central lumen. Triplicate dishes were prepared for each experiment. Values were expressed as number of glandular structures per number of cell colonies. After four days, collagen gels were fixed with 10% neutral buffered formalin for 24 h, removed from the dishes, embedded in paraffin for 4 µm histological sections and stained with haematoxylin/eosin.

*Collagen binding assay*

Microtitre plates (Dynatech) were coated with 50 µl/well of human type-I collagen (Sigma), human type IV collagen (Sigma), mouse laminin (Collaborative Research) and bovine serum albumin (Sigma) at serial concentrations (5, 10, 20, 40 µg ml$^{-1}$) and left uncovered in a laminar flow hood overnight to allow normal evaporation. The plates were then rinsed with phosphate buffered saline (PBS) and used in the binding assay. Trypsinised cells were washed three times in serum-free DMEM and resuspended with DMEM, 2.5 mg ml$^{-1}$ BSA with serial concentrations of HECD-1 and mAb13 monoclonal antibodies. Approximately $5 \times 10^4$ cells per well were plated into previously coated 96-well Dynatech plates and allowed to attach for 1 h at room temperature. The supernatants were then removed and the unattached cells were washed away three times with PBS. The attached cells were fixed with 3% paraformaldehyde and stained with 0.5% toluidine blue in 3.7% paraformaldehyde. Cell attachment was estimated from absorbance measurements at 580 nm performed using an ELISA reader (Minireader II; Dynatech Labs, Inc., VA). Preliminary experiments had shown that the maximal attachment (60%) of SW1222 cells to collagen coated plates was reached after 1 h incubation (data not shown).

*Cell-cell adhesion assay*

This was performed as described by Benchimol *et al.* (1990). Briefly a single cell suspension of SW1222 cell line was obtained by 3 min incubation at 37°C with 0.12% Bacto trypsin in PBS. After centrifugation, the cells were put through a 30-gauge needle in DMEM plus 0.8% FCS. A suspension of $3 \times 10^6$ cells in 3 ml DMEM in 30 ml polystyrene tubes was magnetically stirred at 37°C in an atmosphere of 5% $CO_2$. The number of single cells was determined using a haemocytometer at time 0 and at 120 min. Duplicate cell suspensions were resuspended in DMEM containing each monoclonal antibody used for the collagen gel experiment (HECD-1, mAb13). In addition PR3B10 monoclonal antibody which recognises carcinoembryonic antigen (CEA) and the non-specific cross-reacting antigen (NCA) (Pignatelli *et al.*, 1990*b*) was used.

*Beta-1 integrin and E-cadherin expression by immunocytochemical staining*

Colorectal carcinoma cell lines were grown on glass slides for immunocytochemical staining. For this purpose $2 \times 10^6$ cells were resuspended in 20 ml culture medium (DMEM/10%FCS) and plated in 9 cm Petri dishes containing autoclaved 4 well 'Multiwell' glass slides [C.A. Hendley Ltd, (Essex)]. Cell adherence and growth on the glass slides appeared identical to that seen in plastic Petri dishes. Cells were cultured on the slides for at least 2 days prior to staining by a standard avidin-biotin-complex indirect immunoperoxidase technique.

*E-cadherin expression by Western blot analysis*

Freshly scraped cells were lysed for 5 min at 96°C with 4% SDS, 5% 2-mercaptoethanol in 1 M Tris-HCl buffer (pH 6.8) followed by centrifugation at 10,000. Aliquots of 50 µg of total cell proteins in 30 µl volume were loaded per lane onto 10% SDS-polyacrylamide gel. After electrophoresis, the samples were electroblotted onto nitrocellulose sheets. The sheets were incubated with 3% bovine serum albumin (BSA) for 30 min and then with HECD-1 monoclonal antibody (20–50 µg ml$^{-1}$) for 1 h at room temperature. After seven washes the sheets were incubated with 1:1000 diluted horseradish peroxidase (HRP)-conjugated rabbit anti-mouse immunoglobulins (Dako, Denmark) for 1 h at room temperature. After further washes, the sheets were stained in Tris buffer (50 mM Tris-Hcl, pH 7.4) containing 1-chloro-4-naphthol.

### Results

*Beta 1 integrin and E-cadherin expression on SW1222 and LS174T cells*

To examine the expression of $\beta_1$ integrins and E-cd SW1222 and LS174T cells were grown in glass slides for 48 h and then stained by avidin-biotin-complex indirect immunoperoxidase technique using specific monoclonal antibodies. In SW1222 cells, both $\beta_1$ integrin chain (mAb13) and E-cd (HECD-1) were highly expressed on the cell membrane with accentuation in regions of cell-cell contacts (Figure 1a and b). The expression of E-cd on SW1222 cells was also confirmed by western blot analysis which showed the specific 120 kDa polypeptide (Figure 2, lane 1).

LS174T, which is a moderately differentiated colon carcinoma cell line with poor intercellular cohesion and no ability to undergo morphological differentiation in collagen gel (Pignatelli & Bodmer, 1989), did not express E-cadherin by western blot analysis (Figure 2, lane 2) and immunostaining (Figure 1c).

*The collagen binding of SW1222 cells is mediated by $\beta_1$ integrins*

SW1222 cells showed specific binding to type I collagen, type IV collagen and to a lesser extent to laminin (Figure 3). The type I collagen binding of SW1222 cells was specifically inhibited by the mAb13 in a dose-dependent manner (Figure 4). The monoclonal antibody to E-cd, as predicted, did not show any specific inhibition of the SW1222 collagen binding (Figure 4).

*E-cadherin is a functional cell-cell adhesion molecule expressed by SW1222 cells*

To examine whether E-cd functions as an intercellular adhesion molecule on SW1222 cells, a cell aggregation assay which measures the ability of single cells to form aggregates

**Figure 1** Expression of the common $\beta_1$ integrin subunit on SW1222 cells **a**, and E-cd on SW1222 **b**, and LS174T cells **c**, by avidin-biotin-complex indirect immunoperoxidase technique (bar = 50 $\mu$m).

**Figure 2** Western blot analysis of SW1222 (lane 1) and LS174T (lane 2) cell extracts stained for E-cadherin with the HECD-1 monoclonal antibody. SW1222 cells express the E-cadherin 120 kDa polypeptide (lane 1) whereas LS174T cells (lane 2) showed no E-cadherin reactivity. The unmarked lane represents the molecular weight markers.

in suspension was used. The aggregation of SW1222 cells was completely inhibited by HECD-1 (E-cd) monoclonal antibody. Interestingly both mAb13 ($\beta_1$ integrin) and an anti-CEA/NCA monoclonal antibody (PR3B10) did not significantly inhibit SW1222 cell aggregation (Figure 5).

*Functional cooperation of $\beta_1$ integrin and E-cadherin in the induction of glandular differentiation*

To examine the functional role of $\beta_1$ integrins and E-cd in the induction of the morphological differentiation of SW1222 cells in collagen gel, cells were resuspended with serial dilutions of mAb13 ($\beta_1$ integrin chain) and HECD-1 (E-cd) monoclonal antibodies. The monoclonal antibody W6/32 which recognises HLA class I antigen (Barnstable *et al.*, 1978) was used as negative control. Each monoclonal antibody was subsequently added to the cultured medium for four consecutive days. The plates were scored every day for glandular structures as described in Materials and methods. SW1222 cells grown in the presence of either HECD-1 (Figure 7a), mAb13 (Figure 7b) formed small non coherent aggregates with ill-defined margins and undifferentiated morphology. The degree of glandular differentiation of SW1222 cells was inhibited up to 61% and 75% by the addition of mAb13 and HECD-1 respectively (Figure 6). No change in the morphology of SW1222 cells was seen by adding the control antibody (W6/32) (Figure 7c).

### Discussion

In malignant neoplasia cells fail to fully differentiate and show reduced adhesiveness to one another which may be an important factor in enabling them to infiltrate surrounding tissues and subsequently to detach and migrate (metastasis) (Fidler & Hart, 1982). This loss of differentiation and adhesion is reflected in the cytological and architectural structures. The morphological assessment of the glandular configuration and evaluation of the preserved polarity where cell apex and base are readily distinguished are the most

**Figure 3** Binding of SW1222 cells to type I collagen (TYPE I), type IV collagen (TYPE IV), laminin (LM) and bovine serum albumin (BSA) using a cell adhesion assay. Trypsinised cells were plated into 96-well Dynatech plates previously coated with each extracellular matrix protein or BSA and allowed to attach for 1 h at room temperature. Non-attached cells were washed away with PBS, and the attached cells were fixed with 3% paraformaldehyde and stained with 0.5% Toluidine blue in 3.7% formaldehyde. Cell attachment was estimated from absorbance measurements at 580 nm performed using an ELISA reader. Data shown represent the mean ± standard deviations of three determinations.

**Figure 4** Inhibition of SW1222 cell attachment to type I collagen by mAb13 ($\beta_1$ integrin subunit). Cells were plated in microtitre wells coated with type I collagen (20 μg ml$^{-1}$) and containing the indicated concentrations of monoclonal antibody mAb13 ($\beta_1$ integrin chain) and HECD-1 (E-cadherin). As negative control no monoclonal antibody was added in some wells. Cell attachment was determined as described in Figure 3.

**Figure 5** Inhibition of SW1222 intercellular adhesion by HECD-1 monoclonal antibody (E-cadherin). A single cell suspension of SW1222 cells (10$^6$ ml$^{-1}$) was magnetically stirred at 37°C and the number of single cells determined after 2 h. Duplicate cell suspensions were resuspended in DMEM containing the following monoclonal antibodies: HECD-1 (E-cadherin), mAb13 ($\beta_1$ integrin chain), PR3B10 (CEA/NCA). As negative control no monoclonal antibody was added to some wells.

**Figure 6** Inhibition of the glandular differentiation of SW1222 cells by HECD-1 (E-cadherin), mAb13 ($\beta_1$ integrin chain) and W6/32 (negative control). Cells were cultured in collagen gel in the presence of each monoclonal antibody (50 μg ml$^{-1}$) for 4 days. The plates were scored every day for glandular structures under a phase contrast Diavert Leitz microscope (objective 32L/0.40). Values are expressed as number of glandular structures per number of cell colonies.

reliable criteria to define the grade of malignancy of colorectal tumours (Jass *et al.*, 1986). Thus colorectal carcinomas can be divided in three histological groups of low grade, average grade and high grade according to the degree of tubular differentiation. This classification reflects the behaviour of the tumour and significantly correlates with survival rate (Halvorsen & Seim, 1988). The molecular basis of glandular differentiation is therefore fundamental to our understanding of neoplastic cell behaviour.

In this study we show that both cell-cell and cell-collagen interactions are required for the induction and maintenance of the glandular differentiation of a colon carcinoma cell line (SW1222) in collagen gel and are primarily mediated by two classes of cell adhesion molecules, E-cd and $\beta_1$ integrins. We have previously shown that the ability of SW1222 cells to undergo glandular differentiation is mediated by binding to collagen I matrix via a specific cell surface receptor (Pignatelli & Bodmer, 1988). Here we demonstrate that the functional collagen receptor mediating the morphological differentiation in 3D-collagen gel is a member of the $\beta_1$ integrin subfamily. The known $\beta_1$ integrin collagen receptors expressed by SW1222 cells are $\alpha_2\beta_1$ (VLA-2) and $\alpha_3\beta_1$ (VLA-3) (Pignatelli, 1990). However, the lack of sufficient amount of $\alpha$ subunit-specific monoclonal antibodies has not allowed us yet to identify the $\beta_1$ integrin molecule mediating the glandular differentiation in collagen gel.

Cell-cell interactions are also important in morphogenesis. Studies with polarised epithelial cells grown in culture have shown that under conditions where there is no cell-cell contact, single cells exhibit a poorly differentiated phenotype

**Figure 7** SW1222 cells grown in collagen gel (day 4) in the presence of **a**, HECD-1 (E-cadherin), **b**, mAb13 ($\beta_1$ integrin) or **c**, an irrelevant antibody (W6/32). Collagen gels were fixed with 10% neutral buffered formalin for 24 h, removed from the dishes, embedded in paraffin for 4 $\mu$m histological slides and stained with haematoxylin/eosin (bar = 50 $\mu$m).

with a non polarised distribution of marker proteins of apical and basal and lateral membrane domains (Sztul *et al.*, 1987). Recently it has become clear that cells express a multitude of cell-cell as well as cell matrix adhesion receptors which may control these complex mechanisms. Cadherins are considered to be important regulators of morphogenesis by their homophilic binding specificity. E-cd seems to mediate the selective epithelial cell adhesion which is required for the induction of glandular differentiation of SW1222. No inhibition of cell-cell interactions was seen using the anti-$\beta_1$ integrin monoclonal antibody (mAb13) and the anti CEA/NCA monoclonal antibody (PR3B10) which have been shown to function as cell-cell adhesion molecules (Larjava *et al.*, 1990; Benchimol *et al.*, 1989). These results are in agreement with previous reports showing that as long as cadherins are functioning, the inactivation of other adhesion systems has little effect on cell-cell adhesion (Duband *et al.*, 1987).

Normal epithelial cells always express high levels of E-cd and $\beta_1$ integrins on the cell surface. However both molecules are either lost or down-regulated in poorly differentiated colorectal as well as other malignant epithelial tumours (Pignatelli *et al.*, 1990*a*; Pignatelli *et al.*, 1991, 1992; Shiozaki *et al.*, 1991). Interestingly in some tumours, there is heterogenous E-cd (Edelman *et al.*, 1989; Shiozaki *et al.*, 1991) and $\beta_1$ integrin expression (Pignatelli *et al.*, 1991, 1992) which is often confined to the cytoplasm with no clear cell surface expression. This obviously implies that in transformed cells still expressing cell adhesion molecules in the cytoplasm, the deregulation of cell-cell and cell-matrix interactions is due to loss of function with subsequent disorganisation of the cytoskeletal filaments which are structurally linked to E-cd (Takeichi, 1991) and integrins (Hynes, 1987).

There is increasing experimental evidence that low expression of E-cd and integrins seen in poorly differentiated tumours plays a major role in their biological behaviour. Frixen *et al.* (1991), have shown that human carcinoma cell lines with a dedifferentiated 'fibroblast-like' phenotype had lost E-cd and were highly invasive in an *in vitro* assay. Furthermore the invasive behaviour of dedifferentiated breast carcinoma cell lines was corrected by transfection with E-cd cDNA. Alternatively the introduction of a plasmid encoding E-cd-specific antisense RNA into noninvasive transformed cells rendered the cells invasive (Vleminckx *et al.*, 1991) consistent with a suppressive invasive role for E-cd. Similarly Giancotti and Ruoslahti (1989) have shown by transfection experiments that the overexpression of the $\alpha_5\beta_1$ integrin in Chinese Hamster Ovary cells reestablishes normal growth control *in vitro* with loss of tumourigenicity in nude mice. However the functional cooperation of E-cd and integrins is not fully understood. It is likely that E-cd mediates the selective adhesion by their homotype binding allowing close contact of epithelial cells. In responsive cells which also express functional $\beta_1$ integrin molecules this will allow them to fully respond to the differentiating effect of extracellular matrix proteins (Pignatelli & Bodmer, 1988). Loss of expression and/or function of E-cd and integrins will therefore allow tumour cells to dedifferentiate and lose cohesiveness, properties which would facilitate invasion and metastasis.

Support for this research was provided by the Medical Research Council and Imperial Cancer Research Fund. M. Pignatelli is a recipient of an MRC Clinician Scientist Fellowship.

We thank Sir Walter Bodmer for providing the monoclonal antibodies PR3B10 anbd W6/32, and Dr Kenneth Yamada for the monoclonal antibody mAb13.

**References**

AKIYAMA, S.K., YAMADA, S.S., CHEN, W.-T. & YAMADA, K.M. (1989). Analysis of fibronectin receptor function with monoclonal antibodies: roles in cell adhesion, migration, matrix assembly, and cytoskeletal organisation. *J. Cell Biol.*, **109**, 863–875.

BARNSTABLE, C.J., BODMER, W.F., BROWN, G., GLAFRÉ, C., MILSTEIN, C., WILLIAMS, A.F. & ZIEGLER, A. (1978). Production of monoclonal antibodies to group A erythrocytes, HLA and other cell surface antigen-new tools for genetic analysis. *Cell*, **14**, 9–20.

BENCHIMOL, S., FUKS, A., JOTHY, S., BEAUCHEMIN, N., SHIROTA, K. & STANNERS, C.P. (1989). Carcinoembryonic antigen, a human tumor marker, functions as an intercellular adhesion molecule. *Cell*, **57**, 327–334.

CARTER, W.G., WAYNER, E.A., BOUCHARD, T. & KAUR, P. (1990). The role of integrin $\alpha_2\beta_1$ and $\alpha_3\beta_1$ in cell-cell and cell-substratum adhesion of epidermal cells. *J. Cell Biol.*, **110**, 1387–1404.

DEL BUONO, R., PIGNATELLI, M., BODMER, W.F. & WRIGHT, N.A. (1991). The role of the arginine-glycine-aspartic acid-directed cellular binding to type I collagen and rat mesenchymal cells in colorectal tumour differentiation. *Differentiation*, **46**, 97–103.

DUBAND, J.-L., DUFOUR, S., HATTA, K., TAKEICHI, M., EDELMAN, G.M. & THIERY, J.P. (1987). Adhesion molecules during somatogenesis in the avian embryo. *J. Cell Biol.*, **104**, 1361–1374.

EDELMAN, S., DAMSKY, C.H., WHEELOCK, M.J. & DAMJANOV, I. (1989). Expression of the cell-cell adhesion glycoprotein cell-CAM 120/80 in normal human tissues and tumors. *Am. J. Pathol.*, **135**, 101–110.

EDELMAN, G.M. & CROSSIN, K.L. (1991). Cell adhesion molecules: implications for a molecular histology. *Annu. Rev. Biochem.*, **60**, 155–190.

FIDLER, I.J. & HART, I.R. (1982). Biologic diversity in metastatic neoplasms: origins and implications. *Science* (Washington DC), **217**, 998–1003.

FRIXEN, U.H., BEHRENS, J., SACHS, M., EBERLE, G., VOSS, B., WARDA, A., LOCHNER, D. & BIRCHMEIER, W. (1991). E-cadherin-mediated cell-cell adhesion prevents invasiveness of human carcinoma cells. *J. Cell Biol.*, **113**, 173–185.

GIANCOTTI, F.G. & RUOSLAHTI, E. (1989). Elevated levels of the $\alpha_5\beta_1$ fibronectin receptor suppress the transformed phenotype of Chinese Hamster Ovary cells. *Cell*, **60**, 849–859.

HALVORSEN, T.B. & SEIM, E. (1988). Degree of differentiation in colorectal adenocarcinomas: a multivariate analysis of the influence on survival. *J. Clin. Pathol.*, **41**, 532–537.

HEMLER, M.E. (1990). VLA proteins in the integrin family: structure, functions and their role in leukocytes. *Annu. Rev. Immunol.*, **8**, 365–400.

HYNES, R.O. (1987). Integrins: a family of cell surface receptors. *Cell*, **48**, 549–554.

HYNES, R.O. (1992). Integrins: versatility, modulation, and signaling in cell adhesion. *Cell*, **69**, 11–25.

HYNES, R.O. & LANDER, A.D. (1992). Contact and adhesive specificities in the associations, migrations, and targeting of cells and axons. *Cell*, **68**, 303–322.

JASS, J.R., ATKIN, W.S., CUZICK, J., BUSSEY, H.J.R., MORSON, B.C., NORTHOVER, J.M.A. & TODD, I.P. (1986). The grading of rectal cancer: historical perspectives and a multivariate analysis of 447 cases. *Histopathology*, **10**, 437–459.

LARJAVA, H., PELTONEN, J., AKIYAMA, S.K., YAMADA, S.S., GRALNICK, H.R., UITTO, J. & YAMADA, K.M. (1990). Novel function for $\beta_1$ integrins in keratinocyte cell-cell interactions. *J. Cell Biol.*, **110**, 803–815.

LEIBOVITZ, A., STINSON, J.C., MCCOMBS, W.B. III, MCCOY, C.E., MAZUR, K.C. & MABRY, N.D. (1976). Classification of human colorectal adenocarcinoma cell lines. *Cancer Res.*, **36**, 4562–4569.

PIGNATELLI, M. & BODMER, W.F. (1988). Genetics and biochemistry of collagen binding-triggered glandular differentiation in a human colon carcinoma cell line. *Proc. Natl Acad. Sci. USA*, **85**, 5561–5565,

PIGNATELLI, M. & BODMER, W.F. (1989). Integrin-receptor-mediated differentiation and growth inhibition are enhanced by Transforming Growth Factor-$\beta$ in colorectal tumour cells grown in collagen gel. *Int. J. Cancer*, **44**, 518–523.

PIGNATELLI, M. & BODMER, W.F. (1990). Integrin cell adhesion molecules and colorectal cancer. *J. Pathol.*, **162**, 95–97.

PIGNATELLI, M., SMITH, M.E.F. & BODMER, W.F. (1990*a*). Low expression of collagen receptors in moderate and poorly differentiated colorectal adenocarcinomas. *Br. J. Cancer*, **61**, 636–638.

PIGNATELLI, M., DURBIN, H. & BODMER, W.F. (1990*b*). Carcinoembryonic antigen functions as an accessory adhesion molecule mediating colon epithelial cell-collagen interactions. *Proc. Natl Acad. Sci. USA*, **87**, 1541–1545.

PIGNATELLI, M. (1990). Control of differentiation in colorectal neoplasia. *PhD thesis*, University of London.

PIGNATELLI, M., HANBY, A.M. & STAMP, G.W.H. (1991). Low expression of $\beta_1$,$\alpha_2$ and $\alpha_3$ subunits of VLA integrins in malignant mammary tumours. *J. Pathol.*, **165**, 25–32.

PIGNATELLI, M., CARDILLO, M.R., HANBY, A.N. & STAMP, G.W.H. (1992). Integrins and their accessory adhesion molecules in mammary carcinomas – Loss of polarisation in poorly differentiated tumours. *Human Pathol.* (in press).

RODRIGUEZ-BOULAN, E.R. & NELSON, W.J. (1989). Morphogenesis of the polarized epithelial cell phenotype. *Science*, **245**, 718–725.

RUTZKY, L.P. (1984). The biology of human colon tumour cells in culture. *Adv. Cell Culture*, **4**, 47–83.

SHIMOYAMA, Y., HIROHASHI, S., HIRANO, S., NOGUCHI, M., SHIMOSATO, Y., TAKEICHI, M. & ABE, O. (1989). Cadherin cell-adhesion molecules in human epithelial tissues and carcinomas. *Cancer Res.*, **49**, 2128–2133.

SHIOZAKI, H., TAHARA, H., OKA, H., MIYATA, M., KOBAYASHI, K., TAMURA, S., IIHARA, K., DOKI, Y., HIRANO, S., TAKEICHI, M. & MORI, T. (1991). Expression of immunoreactive E-cadherin adhesion molecules in human cancers. *Am. J. Pathol.*, **139**, 17–23.

STAMP, G.W.H. & PIGNATELLI, M. (1991). Distribution of $\beta_1$, $\alpha_1$, $\alpha_2$ and $\alpha_3$ integrin chains in basal cell carcinomas. *J. Pathol.*, **163**, 307–313.

SZTUL, E.S., BIEMESDERFER, D., CAPLAN, M.J., KASHGARIAN, M. & BOYER, J.L. (1987). Localization of Na+, K+-ATPase subunit to the sinusoidal and lateral but not canalicular membranes of rat hepatocytes. *J. Cell Biol.*, **104**, 1239–1248.

TAKEICHI, M. (1988). The cadherins: cell-cell adhesion molecules controlling animal morphogenesis. *Development*, **102**, 639–655.

TAKEICHI, M. (1991). Cadherin cell adhesion receptors as a morphogenetic regulator. *Science*, **251**, 1451–1455.

VLEMINCKX, K., VAKAET, L. Jr, MAREEL, M., FIERS, W. & VAN ROY, F. (1991). Genetic manipulation of E-cadherin expression by epithelial tumor cells reveals an invasion suppressor role. *Cell*, **66**, 107–119.